# Sealing mechanism study of laryngeal mask airways via 3D modelling and finite element analysis

**DOI:** 10.1038/s41598-022-06908-y

**Published:** 2022-02-21

**Authors:** Hongxia Liao, Liqiang Chen, Meiling Liu, Junfeng Chen

**Affiliations:** 1 Present Address: Department of Anaesthesia, Shanghai Shibei Hospital, Shanghai, 200435 China; 2 Present Address: Department of Surgery, Shanghai Shibei Hospital, Shanghai, 200435 China

**Keywords:** Health care, Medical research

## Abstract

Proper sealing of laryngeal mask airways (LMAs) is critical for airway management in clinical use. Understanding the sealing mechanism can significantly help front-line anaesthetists to reduce the incidence of adverse events. However, anaesthetists, who may not have the most substantial engineering backgrounds, lack intuitive ways to develop an understanding of the LMA sealing mechanism effectively. The paper aims to study the LMA-pharynx sealing mechanisms from the perspective of front-line anaesthetists. We use a computer-aided 3D modelling technique to visualise the LMA—pharynx interactions, which helps anaesthetists identify the critical areas of complications. Furthermore, we conduct a quantitative pressure distribution analysis of the LMA-pharynx contacting surface using the finite element analysis technique, which helps further understand the sealing mechanics in those areas. We present two cases studies based on one male volunteer, aged 50, inserted with a ProSeal LMA. In the first case, a relatively low cuff pressure (CP) was applied to simulate the clinical circumstances in which complications related to air leakage are most likely to happen; in the second case, we increase the CP to a relatively high value to simulate the scenarios with an increased risk of complications related to high mucosal pressure. The experiments suggest the follows: (1) Sore throat complications related to high mucosal pressure is most likely to occur in the hypopharynx with a high CP setting, particularly in the areas where the cricoid cartilage presses the mucosa. (2) The narrow hyoid bone super horn width likely causes LMA insertion difficulties. (3) Insufficient CP will significantly increase the risk of air leakage in the oropharynx. A complete sealing pressure line in the contacting surface will be formed with sufficient CP, thereby preventing the air leakage into the oral.

## Introduction

Laryngeal mask airways (LMAs) are widely adopted supraglottic airway devices in airway management of operations and first aid treatments. Compared with other airway management methods, e.g., endotracheal tube ventilation, the LMA has various advantages, including simple-to-use, stable circulation, fast recovery, and fewer complications^1–3^. Therefore, LMAs have gradually become one of the most popular artificial airways.

**Figure 1:**
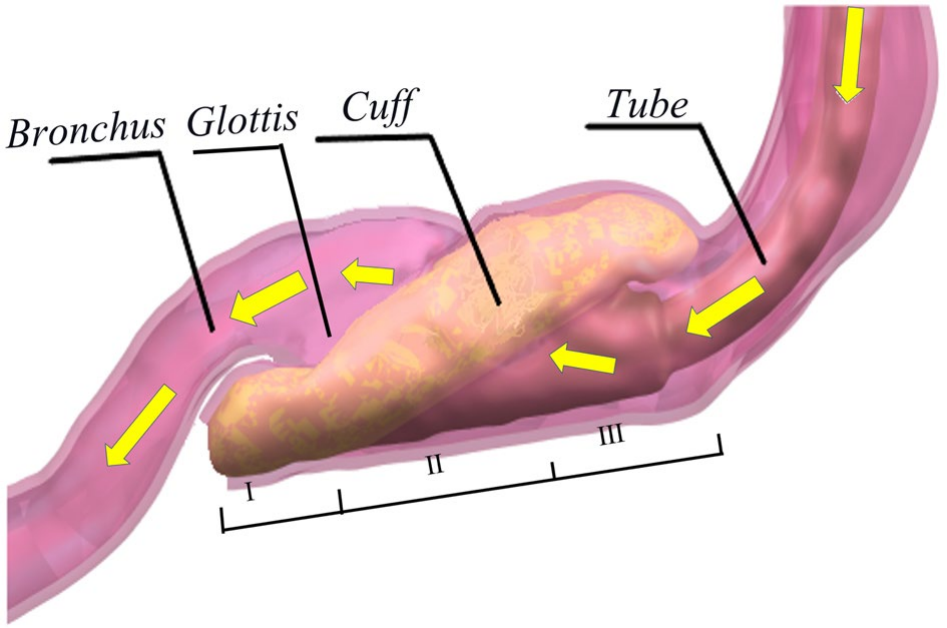
Pharynx with an inserted LMA (hypopharynx (I); laryngopharynx (II); oropharynx (III); yellow arrows indicating the airflow direction during positive pressure ventilation).

To be self-contained, here we briefly describe the working principle of LMAs. When an LMA is inserted into the laryngopharynx for ventilation, oxygen successively passes through the ventilator, the tube of LMA, the cuff of LMA, the glottis and the bronchus, respectively, and finally enters the lung, as indicated by the yellow arrows in Fig. 1. The inflated cuff interacted with the oropharynx and the hypopharynx, forming a sealing ring around the glottis to ensure sustainable airflow from the ventilator to the lung. Therefore, a good sealing LMA to the pharynx, particularly the hypopharynx and the oropharynx, is critical to a successful operation.

According to the current clinical protocol, anaesthetists need to determine the right LMA size based on the manufacturer’s guidance. Most LMA manufacturers adopt weight-based criteria, i.e., to choose the LMA size based on patients’ bodyweight. However, the size of the airway anatomy is not always proportional to bodyweight. As documented in^4,5^, the weight-based selection criteria may not lead to a satisfactory success rate of LMA insertion attempts.

In clinic practise, anaesthetists can resolve some position and sealing issues after selecting an LMA by adjusting the CP value based on their experience. However, an under-inflated LMA may not entirely block the oropharynx and the hypopharynx, leading to air leakage, whilst cuff hyperinflation will exert high forces on pharynx structures, and can lead to severe complications in the upper airway, thus impairing mucosal perfusion^6–9^. Recent studies indicate that various factors, including volume of LMAs^10^, pressure on the pharyngeal mucosa^11^ and ventilation modes^12^, can significantly influence the sealing performance. Some studies^4,13^ suggested that cricoid cartilage also had a particular effect on the ventilation with LMAs, but few studies on the impact of other factors. Overall, there is a lack of consensus on the causes of air leakage and high MP, and few guidelines for front-line anaesthetists to resolve complications due to a lack of knowledge of the LMA sealing mechanism.

Early studies on LMA sealing mechanisms^14^ adopted direct measurement methods by installing additional measurement electronics, including stethoscopes, end-tidal $\hbox {CO}_2$, and aneroid manometers. Despite their abilities to directly obtain the oropharyngeal leakage pressure at certain locations, these methods cannot measure the MP or detect the specific leakage sites of an LMA, which are the main factors causing complications. To overcome the limitation of those methods, Brimacombe et al.^15^ implanted a pressure sensor to an LMA to measure the MP and the CP. In the meantime, the colour of the pharyngeal mucosa was observed as an indicator of the perfusion pressure. In this manner, the stress-strain correlation can be established between the pressure inside the laryngeal mask and the mucosal perfusion pressure. Although those electronic-based methods have demonstrated their efficacy in studying the LMA sealing at the preselected measurement points, e.g., piriform fossa, lateral pharyngeal wall, it would be challenging to help front-line anaesthetists develop a big picture of the sealing process. This is because only a limited number of measurement points can be picked in the electronics-based study, e.g., piriform fossa, lateral pharyngeal wall. In other words, those methods do not provide an entire picture for front-line anaesthetists on how the LMA interacts with the pharynx during operation.

This paper studies the LMA-pharynx sealing mechanism from anaesthetists’ perspective. Instead of focusing on some preselected points, this work provides a complete visualisation on the LMA-pharynx interaction and qualitative analysis on the pressure distribution of LMA-pharynx contacting surface, i.e., a comprehensive understanding of the sealing mechanism. This understanding will help the anaesthetists in clinical practice, including choosing the proper size of LMA, adjusting to an appropriate CP to prevent air leakage, resolving LMA insertion difficulties, etc.

Instead of relying on direct measurements requiring extra hardware like the electronics-based methods^14^, this work employs computer-aided 3D modelling and finite element analysis (FEA) techniques to investigate the sealing mechanism of an LMA. Computer-aided modelling and FEA have demonstrated their efficacy in numerous engineering problems^16,17^. Recently, substantial research has been devoted to applying computer-aided modelling and FEA techniques to study biomechanics and predict biomechanical phenomena^18–21^. A heart and arterial finite element model was established^22^ to simulate aortic blood flow and explore the vessel wall dynamics. Ref.^23^ applied FEA on the mechanics of shear and tensile stresses in the articular cartilage and investigated the possibility of causing potential cartilage pathologies, such as osteoarthrosis. Ref.^24^ applied FEA to study the airflow-lung lower airway interactions using the partitioned fluid-structure interaction approach. FEA techniques have also demonstrated efficacy in studying the sealing mechanism for mechanical structures^25^. However, to the best of the authors’ knowledge, no existing research has adopted 3D modelling and FEA techniques to provide an intuitive way to understand the LMA sealing mechanism for anaesthetists, who may not have the most substantial engineering backgrounds.

The study process of this paper can be summarised as follows:

- Firstly, we establish a set of 3D models of the LMA-pharynx interactions. The 3D models not only provide intuitively global visualisation, but also help identify critical areas where air leakage and high MP problems may happen.
- Secondly, we exploit finite element analysis on a pressure distribution of the LMA-pharynx contacting surface, which helps anaesthetists understand complete sealing details, particularly critical areas.

This novel approach resolves the limitations of the existing methods and can help anaesthetists develop an intuitive understanding of the interactions between the pharynx and the LMA. The experimental results support the following findings:

- Sore throat complications caused by high MP are mostly like to happen in the hypopharynx in high CP settings, particularly where the cricoid cartilage presses the mucosa.
- The narrow hyoid bone super horn width mostly causes LMA insertion difficulties. We suggest anaesthetists to take the super horn width into consideration when selecting the LMA size.
- Insufficient CP will significantly increase the risk of air leakage. We suggest anaesthetists adjust the CP carefully so that a complete sealing pressure line will be formed in the oropharynx to prevent air leakage.

The remainder of the paper is organised as follows. The computer-aided 3D modelling process and the details of FEA settings are described in “Methods” section. In “Results and discussion” section presents the results and the discussions. Finally, the paper is concluded in “Conclusion” section.

## Methods

We construct a set of 3D models using Computerised Tomography (CT) to directly visualise the internal structure of LMA-pharynx interaction from different directions. From the 3D models, we can get an intuitive understanding of the sealing situations, which helps us identify critical areas with a high risk of complications caused by high MP and air leakage. Next, FEA is carried out to give a quantitative study on the sealing mechanics on the contacting surface in the critical areas. The results can help us develop a comprehensive understanding of the essential physics of the sealing mechanism.

We build a neck model by considering the pharynx and bones around it as a whole to investigate the interaction between the neck and LMA. We refer to the Pharynx and Bones as “PB” in the following content. The 3D models are established based on the following ideas:

- As PB provides supporting pressure that seals pharyngeal mucosa to the LMA, we simplified the neck model as a combination of pharynx and bones around it (PB model).
- To study sealing mechanics and the sealing the cause of complications, we created two PB-LMA models, including one model of PB with LMA loaded with a relatively low CP, referred to as “LMA50”; and another PB model with LMA loaded with a high CP, referred to as “LMA100”, respectively.

Two case studies are presented based on an LMA inserted into a volunteer. In Case I, the LMA is deliberately loaded with a low CP (9 mmHg) to investigate the cause of leaking. In Case II, the CP is increased to 45 mmHg, a recommended value by the manufacturer to prevent air leakage, to detect the critical points of high MP.

### Data source

A volunteer (male, 50 years old, 71.2 kg, 171 cm, ASA (American Society of Anesthesiologists) class I^26^, no underlying conditions) were recruited for this study. A ProSeal^27^ LMA of size 5 was placed after induction of anaesthesia, and a manometer measured the CP inside the cuff simultaneously. Written informed consent was obtained from volunteers, and research was approved by the Ethics Commission of Shibei Hospital of Jingan District, Shanghai, China, with an ethical report. All of the methods used in this study were carried out per the relevant guidelines and regulations^28^.

### 3D modelling of LMA-pharynx interactions

A 64-slice spiral CT (Philips, Ingenuity CT, Israel) is used to obtain the image data of the PB-LMA model. The scanning process is detailed as follows. Firstly, topical anaesthesia is carried out adequately on the patient’s throat. Next, intravenous anaesthesia is carried out for the patient with the monitor of vital signs. CT scans are carried out to obtain data of PB with the inserted LMA loaded with different CP values, measured by a manometer at the LMA inflation valve.

**Figure 2:**
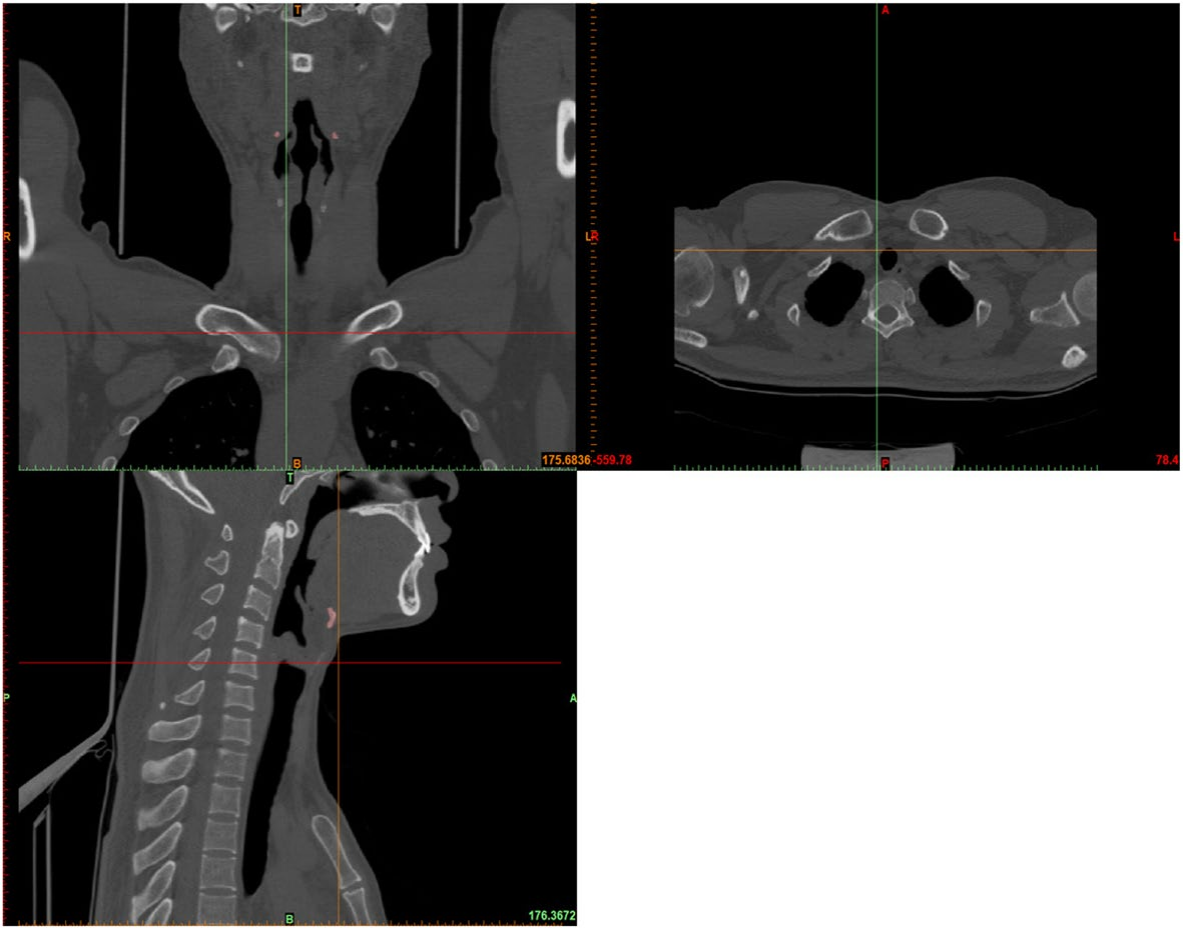
CT image displayed in the GUI of 3D modelling software from different views.

**Figure 3:**
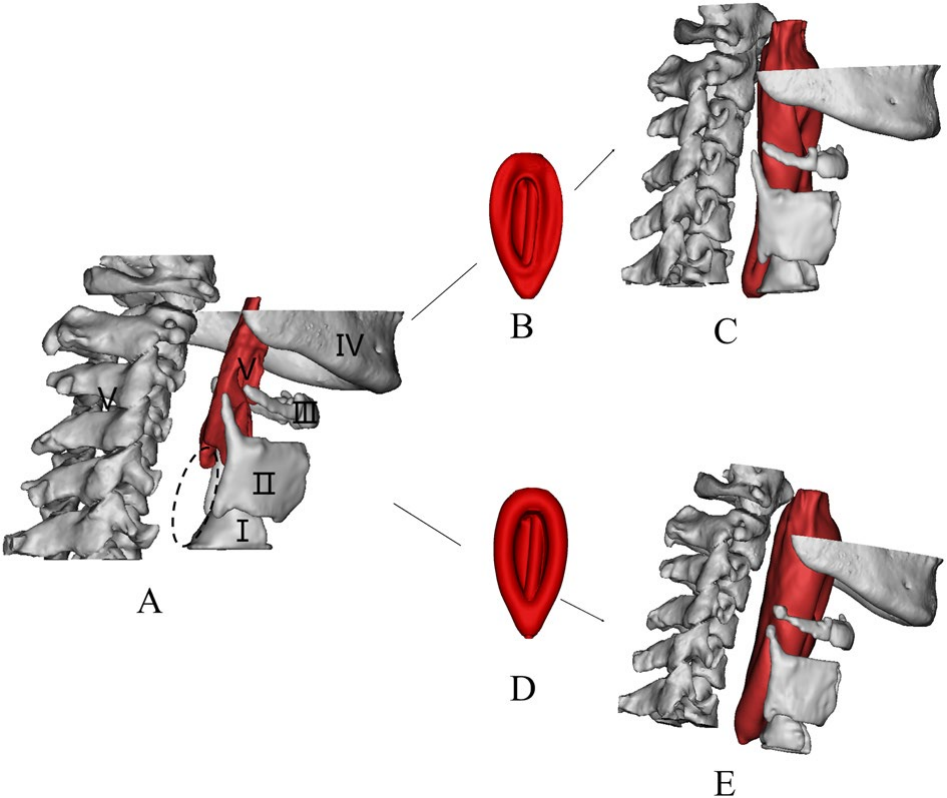
3D reconstruction model of PB and LMA (A: 3D image of PBs, including cricoid cartilage (I), thyroid cartilage (II), hyoid bone (III), mandible (IV), spine (V), B: LMA50; C: 3D image of PB-LMA50; D: LMA100; E: 3D image of PB-LMA100. The position indicated by the elliptical dotted line is the closed hypopharynx).

The scanning parameters are detailed as follows: 120 kV, 30 mAs, 1 mm scanning thickness, 0.75 mm $\times$ 0.75 mm in-plane resolution, $512 \times 512$ matrix. Firstly, CT images in DICOM format obtained for each scan, as displayed in Fig. 2, are used as references to establish 3D models of PB-LMA interactions. After threshold segmentation, dynamic region growth, 3D calculation and smoothing, we constructed 3D models for each part of the PB-LMA interactions are, as shown in Fig. 3.

### FEA settings

Since each PB part has different mechanics characteristics, we disassemble the PB model into a hyoid bone model, two cartilages (thyroid cartilage and cricoid cartilage), pharyngeal cavity, and muscle around the pharynx. Materials of PB-LMA models include silica gel, silicone, laryngeal soft tissue, muscle, hyoid bone etc. In this study, we define the properties based on studies^29–31^ and data provided by the manufacturer. The related parameters are summarised in Table 1.

**Table 1:** Element type and material parameters.

Structure	Materials	Elastic modulus (MPa)	Poisson ratio ($\mu$)
Cuff	Silicone	6	0.47
Tube	Rubber	10	0.43
Pharyngeal cavity	Soft tissue	13	0.4
Muscle around pharynx	Soft tissue	13	0.4
Hyoid bone	Cortical bone	12,000	0.3
Thyroid and cricoid cartilages	Cartilage	60	0.4

Next, we set the boundaries in terms of the contacting behaviour between components. For example, we set the contact constraints between cuff and tube as binding constraints to simulate the physical connection. Similarly, interactions between the bone tissue and the pharyngeal muscles were also selected as binding constraints. The contacting surface between the cuff and the pharynx were set as friction constraints. Due to the presence of lubricant in pharyngeal mucosa, these friction constraints’ friction coefficients were set to 0.1 to simulate their physical contact behaviour. Following similar philosophies, the end of the tube, bones, and the end of the trachea were fixed support to model the supporting effect of the body and LMA fixing device.

**Figure 4:**
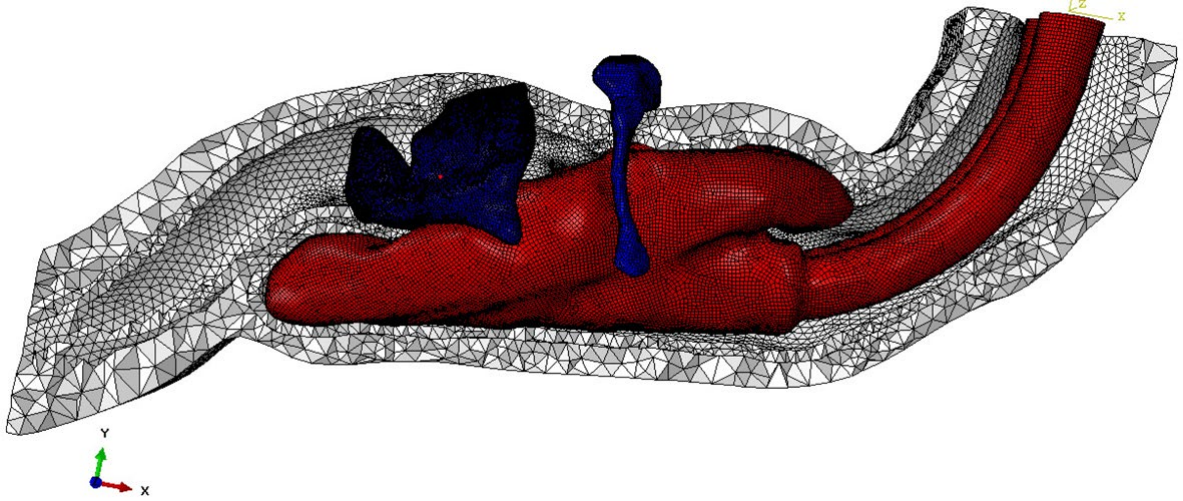
The inserted LMA is divided into shell elements, and PBs are divided into tetrahedron elements.

In biomechanics, human tissues are usually in complex irregular geometry. To perform the FEA calculation efficiently, we divide the 3D PB-LMA models into high-quality tetrahedral mesh instead of hexahedral mesh commonly used to calculate the regular geometric model. The meshing principle is to increase the meshing density at the place of a mutual contact or stress concentration. The meshing is adjusted by trial and error, and the final meshing results are shown in Fig. 4. We can see the meshing density near the contacting surface is significantly higher than other parts.

The loads used in this experiment match the CP values measured at the CT scanning step during clinics, which simulate one high CP scenario to study the high MP phenomenon, and one low CP scenario to study the air leakage as described in “2” section. The data of thickness of the cuff film were provided by the manufacturer, whose details are provided in Table 2.

After finishing the settings, we complete the FEA calculations using ABAQUS software^32^ , on a standard workstation with the following configurations: Dual CPU, each CPU has a dominant frequency of 3.33 GHz, a total of 24 cores and 48 threads; memory: 48 GB; graphics card: 5 generations of core display; bit width: 256 Bit, video memory: 2 GB. The computational time is 40 minutes, which indicates that the FEA can be effectively calculated.

**Table 2:** FEA loading parameters.

Size of the LMA	Thickness of cuff membrane	CP (LMA100)	CP (LMA50)
5#	0.7 mm	45 mmHg	9 mmHg

## Results and discussion

This section presents the results in two parts. Firstly, 3D models are presented to directly visualise the PB-LMA interactions, providing intuitions of critical areas of air leakage and high MP. Secondly, FEA results are presented with a specific focus on PB-LMA interactions in those essential areas to understand the seal mechanics comprehensively.

### 3D visualisation

**Figure 5:**
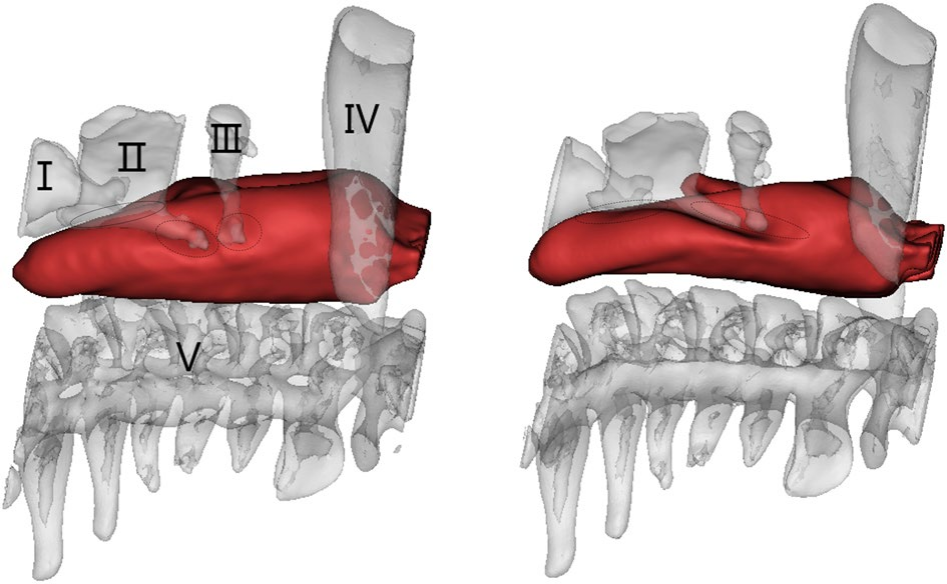
The 3D image of PB-LMA100 (left) and PB-LMA50 (right)( the cricoid cartilage (I), the thyroid cartilage (II), the hyoid bone (III), mandible (IV), the spine (V), and the mucosa inserted with an LMA).

The 3D geometrical models of the PB-LMA100 and PB-LMA50 are shown in Fig. 5, including the cricoid cartilage (I), thyroid cartilage (II), the hyoid bone (III), the mandible (IV), and the spine (V), respectively. When LMA is inflated, bones I–V push mucosa to the inserted LMA, ensuring a proper sealing performance.

**Figure 6:**
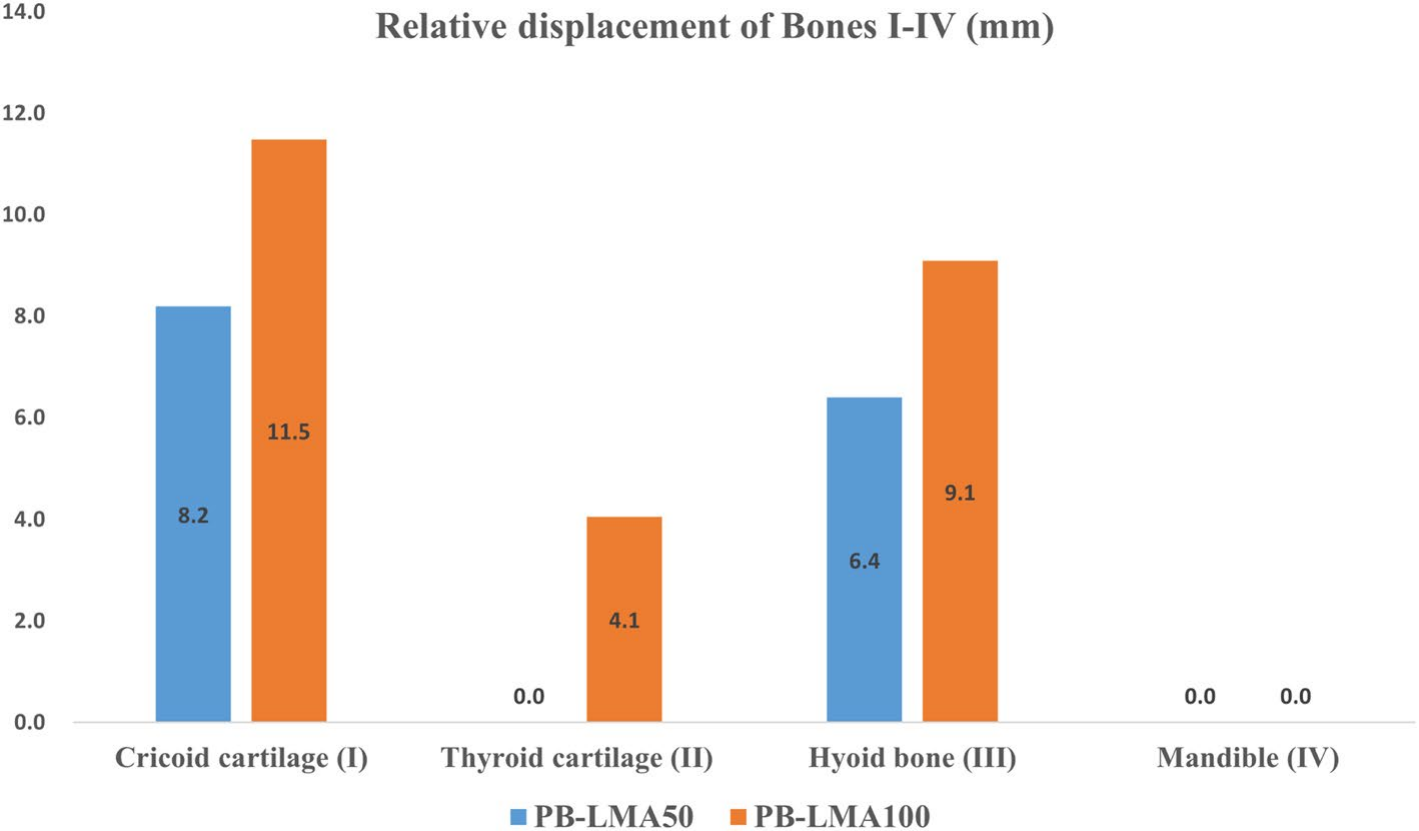
Displacement of pharyngeal Bones I–IV caused by LMA inflation, with respect to the spine (V).

Figure 6 shows the relative displacements of bones I–V after inflating the LMA cuff, respectively, with respect to the spine (V). This can be viewed as an indicator of the force acting by bones I–V on the mucosa. We can see from Fig. 6 there is almost no displacement of the mandible, even when the LMA is loaded with a high CP. This implies very little support is acted on mucosa from the mandible, indicating a possible critical air leakage point. There are significant displacements on the cricoid cartilage (I), the thyroid cartilage (II), and the hyoid bone (III), especially when a high LMA CP is applied. This implies Bones I–III could be potential critical points of high MP. These findings can also be verified by observing of 3D models in Fig. 5, where few folds appear around the mandible (IV) while several notches around Bones I–III.

This subsection gives qualitative results of the sealing situations on several positions. FEA results will be presented in the following subsection to provide a more comprehensive quantitative study of PB-LMA interactions, especially on those critical points.

### FEA MP distribution results

This subsection provides a detailed quantitative analysis to provide a more comprehensive analysis of sealing mechanics. To better demonstrate the LMA-pharynx interactions, we present MP distribution diagrams after completing the FEA calculation. Based on the separation of the pharynx as defined in Fig. 1, the MP distribution diagrams are reorganised into three groups

- MP distributions in the hypopharynx, where the primary interactions are between Bones I–II and the mucosa;
- MP distributions in the laryngopharynx, where the primary interactions are between Bones II–III and the mucosa;
- MP distributions in the oropharynx, where the primary interactions are between Bone IV and the mucosa.

**Figure 7:**
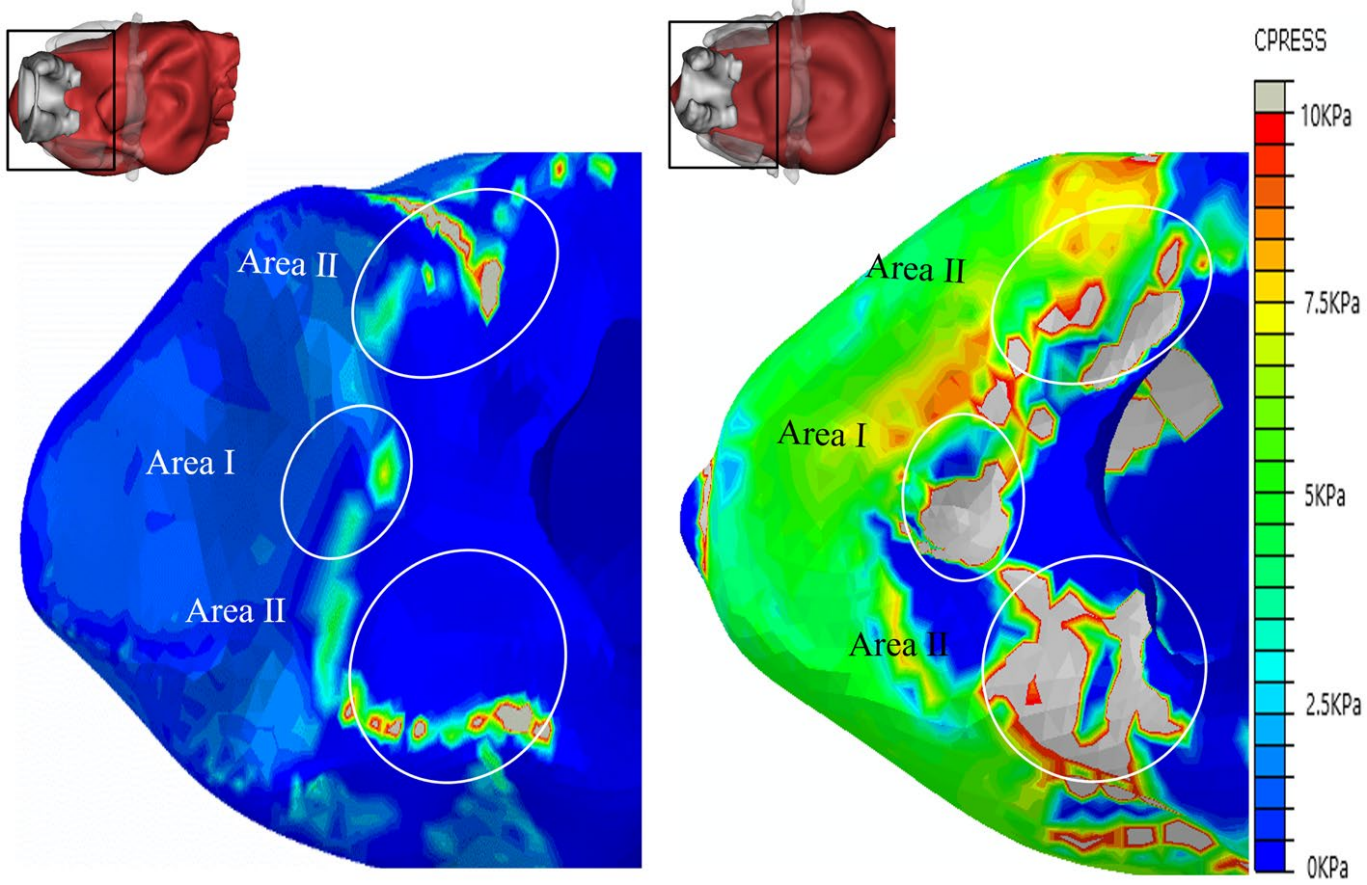
The pressure on hypopharyngeal mucosa of PB-LMA50 (left) and PB-LMA100 (right). Area I shows the impact of the cricoid cartilage (Bone I) pressing the mucosa from the top; Area II shows the impact of the thyroid cartilage (Bone II) squeezing the mucosa from the sides. The peak MP magnitudes are 7.5 kPa (Area I, 9 mmHg), 49.9 kPa (Area I, 45 mmHg), 26.1 kPa (Area II, 9mmHg) and 114.7 kPa (Area II, 45 mmHg), respectively.

#### Hypopharynx

Figure 7 shows the pressure distribution of hypopharyngeal mucosa when the CP values of LMA are 9 mmHg (PB-LMA50, left) and 45 mmHg (PB-LMA100, right), respectively. We can see that in both cases, the high MP points appeared in Area I, where the cricoid cartilage (Bone I) presses the mucosa from the top, and Area II, where the thyroid cartilage (Bone II) squeezes the mucosa from sides.

As the LMA CP increased from 9 to 45 mmHg, the peak MP magnitudes in Areas I–II increased significantly, e.g., from 7.5 to 49.9 kPa in Area I, and from 26.1 to 114.7 kPA in Area II. This is because Bones I–II limits the hypopharynx volume, as observed from the 3D models presented on the left top corners 7. When LMA is inflated, Bones I–II introduce serious reactive effects, leading to high MP in those Areas.

We adopt an indirect verification approach since we cannot use the electronics to verify the pressure distribution at all critical points in this study for ethical considerations. Ref.^33^ studied the effect of LMA CP on postoperative sore throat incidence. The chance of sore throat complication reports increases as the increase of CP value. This also matches our experience in the daily clinical practice at the Department of Anaesthesiology, Shanghai Shibei hospital. Moreover, we also found that the LMA CP positively relates to high blood pressure during operation. Thus, we use postoperative sore throat incidence reports and intraoperative blood pressure to verify our results. Our volunteer complained of sore throat incidence, and the intraoperative blood pressure increased, which indirectly verified the results.

#### Laryngopharynx

**Figure 8:**
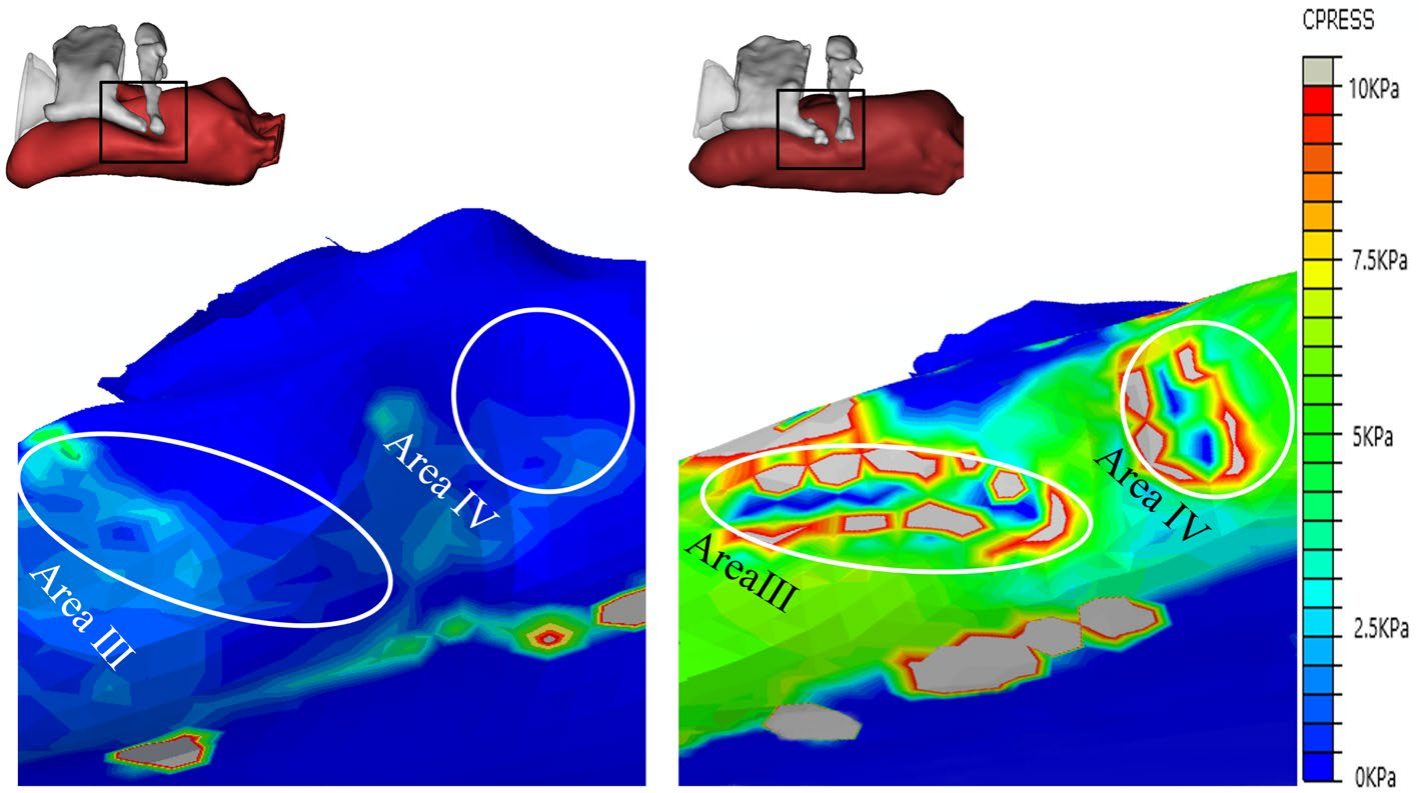
The pressure on laryngopharynx mucosa of PB-LMA50 (left) and PB-LMA100 (right). Areas III–IV show the impact of the super horns of the thyroid cartilage (Bone II), and the hyoid bone (Bone III), squeezing the mucosa from the sides. The peak MP magnitudes are 5.2 kPa (Area III, 9 mmHg), 21.7 kPa (Area III, 45 mmHg), 3.9 kPa (Area IV, 9 mmHg) and 14.9 kPa (Area IV, 45 mmHg), respectively.

Figure 8 shows the CP distribution on the laryngopharyngeal mucosa when the LMA CP values are 9 mmHg (PB-LMA50, left) and 45 mmHg (PB-LMA100, right), respectively. Firstly, by comparing the results in Fig. 8 with Fig. 7, we can see that the MP in the laryngopharynx is much smaller than in the hypopharynx. When the LMA is loaded with 45 mmHg CP, two high MP Areas III–IV appear on both sides of the LMA, located at the areas of the hyoid bone and the super corner of the thyroid cartilage. The peak MP magnitudes are roughly 1/5 to 1/8 of the peak MP in Area I. This is because the laryngopharynx less restricted the inserted LMA than the hypopharynx.

Secondly, as shown in Fig. 8, the super horns of the thyroid cartilage (Bone II) and the hyoid bone (Bone III) form a U-shape, providing support to LMA from both sides, which help keep the LMA in the proper insertion position. This also matches our daily clinical experience at the department of anaesthesiology, Shanghai Shibei Hospital. The LMA size selection needs to consider the width of Bone II and Bone III, because Bones II and III will block an oversized LMA from reaching the targeted position, whilst Bones II and III may not provide enough support to keep an undersized LMA in the ideal place after insertion.

#### Oropharynx

**Figure 9:**
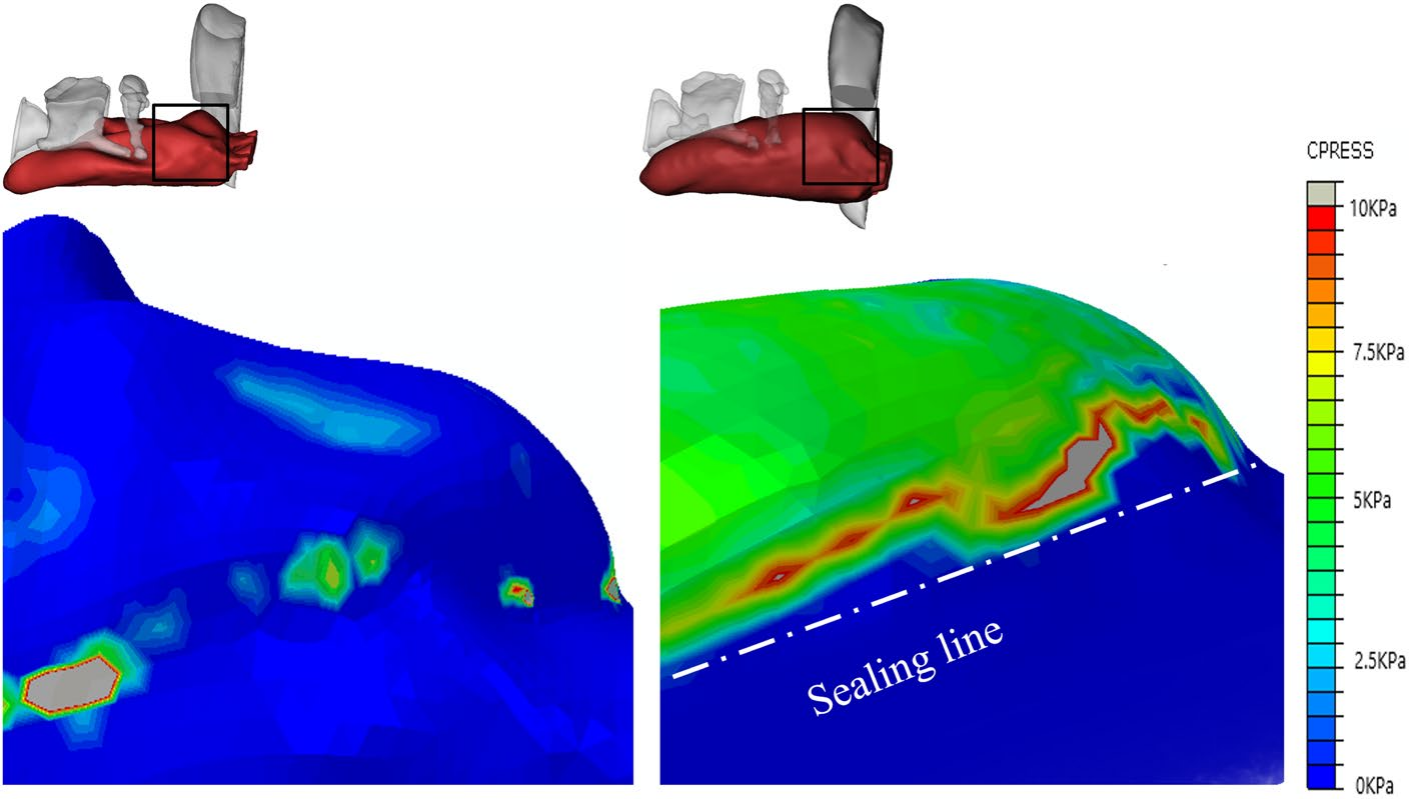
The pressure on oropharyngeal mucosa of PB-LMA50 (left) and PB-LMA100 (right) (lateral view). The pressure of most oropharyngeal mucosa is almost 0 kPa in PB-LMA50; the white dash-dotted line in left image means a line sealing pressure is developed when the cuff pressure increased to recommended value.

**Figure 10:**
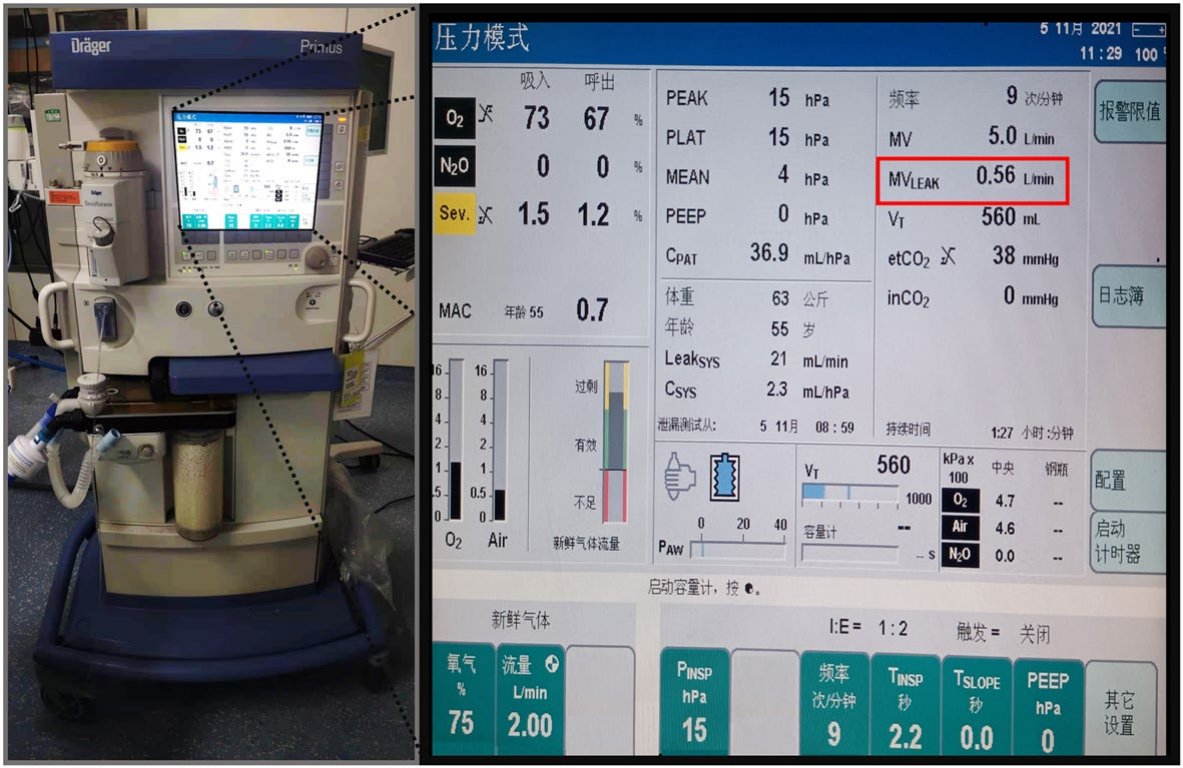
The air leakage during LMA ventilation is detected by the Primus anaesthesia machine. (The red box shows the leakage value).

Figure 9 shows the pressure distribution of oropharyngeal mucosa when the inserted LMA is loaded with a 9 mmHg CP (PB-LMA50, left), and a 45 mmHg CP (PB-LMA100, right), respectively. We can see that when the CP is 9 mmHg, the MP value is close to 0 in most part of the oropharynx, which implies very little pressure support from PB in the oropharyngeal area to seal the mucosa to LMA, leading to a high risk of air leakage in LMA ventilation. The air leakage phenomena have been monitored by the Primus anaesthesia machine^34^. A photo of detecting air leakage in daily practice using the anaesthesia machine is shown in Fig. 10. Meanwhile, we have also scanned the pharynx with an ultrasonic high-frequency probe. The leakage location has also been found from cuff film vibration under airflow, which also aligns with our results.

The air leakage problem is significantly reduced when the CP value is increased to 45 mmHg. A line of sealing pressure support is provided by the PB (the white dash line in the right subfigure of Fig. 8) along the contacting surface between the oropharyngeal mucosa and the inserted LMA, to prevent the air leakage into the oral. We also notice that the peak MP is 10 kPa, indicating high MP to be a less prominent problem in the oropharynx than in other parts of the pharynx.

### Discussion

It can be seen that the FEA results highly align with visualisation results and provide qualitative details on the sealing mechanics. The results obtained from the previous two subsections support the following:

- Air leakage is most likely in the oropharynx, especially when the LMA is loaded with low CP. This is because the mandible (IV), as part of the skull, has a relatively large distance to the spine, providing little supporting pressure to the soft tissues in this space to seal the mucosa to the inserted LMA. However, as we increase CP to the recommended value by the manufacturer, a line of sealing pressure is developed, as shown in Fig. 9, which remarkably improves the sealing performance in preventing air leakage into the oral.
- The high MP areas correspond to the parts where PB directly interacts with the LMA. As shown in Fig. 3, the inserted LMA is jointed supported by (1) the spine (Bone V) from the bottom, (2) the cricoid cartilage and the thyroid cartilage (Bones I–II) from top-left, (3) the super horns of the thyroid cartilage and the hyoid bone (Bones II–III) from the sides, and the LMA tube from the right. The friction effect is negligible due to the lubricant effect of mucus.
- The hypopharyngeal MP has a significantly higher chance of causing soar throat complications than the laryngopharyngeal MP. This is because the gastrointestinal sphincter, which prevents gastric acid reflux in daily life, pulls the cricoid cartilage (Bone I) firmly to the spine. This results in a substantial squeezing effect after the LMA insertion, leading to the high hypopharyngeal MP.

These findings are also reflected in our clinical practices at the Department of Anaesthesia, Shanghai Shibei Hospital. In line with the findings above and our practical clinical experience, we carefully suggest the followings

- Anaesthetists should pay attention to the width of hyoid bones to select a proper LMA size. It would also help if the LMA suppliers can provide LMAs with more selections.
- The CP must be gradually increased to a proper value that the air leakage is just avoided, i.e. the minimum CP avoiding air leakage. This helps to reduce the complication risk caused by high MP.
- The shape of LMA plays a crucial part in LMA sealing performance. To avoid an inevitable trade-off between high MP and air leakage, we suggest the LMA manufacturers provide more specific designs for targeted patient groups.

## Conclusion

This work has developed a novel method for investigation of LMA sealing mechanism by using computer-aided 3D modeling and FEA techniques. Compared with the existing methods, our method does not introduce any additional hardware costs and is capable of providing globe monitoring on the LMA and detailed understanding of sealing mechanics on all areas. A set of 3D models have been established based on CT data for directly visualising LMA-pharynx interactions, identifying the critical areas where air leakage and high MP occur. Furthermore, FEA technique is adopted to provide quantitative analysis on MP distribution in those crucial areas.

It has been found that the FEA results are highly in line with visualisation results and provide qualitative details of the sealing mechanics on the critical points. The experimental results support the following findings: (1) Air leakage is most likely to occur in the oropharynx; (2) High MP is most likely to occur in hypopharnx; (3) Insertion difficulties are usually caused by narrow hyoid bone width. Based on the findings and our practical clinical experience, corresponding suggestions and guidelines are also provided to anaesthetists.

Due to the ethnic and resource limitations, we were not able to validate the correctness of computational results with a direct measurement. Instead, we validated the results through the clinical observations and the patient’s responses. In the future, we would apply for permissions to recruit more volunteers and to carry out electronics-based experiments as the direct validation of the results.

## Abbreviations

3D: Three dimensional
FEA: Finite element analysis
LMA: Laryngeal mask airway
CP: Cuff pressure
MP: Mucosal pressure
PB: Pharynx and bones
CT: Computerised tomography
GUI: Graphical user interface
ASA: American society of anesthesiologists
