# Author Correction: Sealing mechanism study of laryngeal mask airways via 3D modelling and finite element analysis

**DOI:** 10.1038/s41598-022-07964-0

**Published:** 2022-03-02

**Authors:** Hongxia Liao, Liqiang Chen, Meiling Liu, Junfeng Chen

**Affiliations:** 1 Present Address: Department of Anaesthesia, Shanghai Shibei Hospital, Shanghai, 200435 China; 2 Present Address: Department of Surgery, Shanghai Shibei Hospital, Shanghai, 200435 China

Correction to: *Scientific Reports* 10.1038/s41598-022-06908-y, published online 21 February 2022

In the original version of this Article, Meiling Liu and Junfeng Chen were omitted as a corresponding author. Correspondence and requests for materials should also be addressed to meilingliu@163.com and drchenjf@126.com.

The original Article has been corrected.

